# The History and Prospects of Rabbit Sperm Sexing

**DOI:** 10.3390/vetsci10080509

**Published:** 2023-08-07

**Authors:** Patrícia Pinto-Pinho, Ana F. Ferreira, Rosário Pinto-Leite, Margarida Fardilha, Bruno Colaço

**Affiliations:** 1 Centre for the Research and Technology of Agro-Environmental and Biological Sciences, University of Trás-os-Montes and Alto Douro, 5000-801 Vila Real, Portugal; bcolaco@utad.pt; 2 Laboratory of Signal Transduction, Institute of Biomedicine, Department of Medical Sciences, University of Aveiro, 3810-193 Aveiro, Portugal; mfardilha@ua.pt; 3 Laboratory of Genetics and Andrology, Hospital Center of Trás-os-Montes and Alto Douro, E.P.E, 5000-508 Vila Real, Portugal; mlleite@chtmad.min-saude.pt; 4 Experimental Pathology and Terapeutics Group, IPO Porto Research Center, Portuguese Institute of Oncology of Porto Francisco Gentil, E.P.E., 4200-072 Porto, Portugal; 5 Animal and Veterinary Research Centre, University of Trás-os-Montes and Alto Douro, 5001-801 Vila Real, Portugal; anafilipa.pferreira@ua.pt

**Keywords:** sperm sexing, cuniculture, sex ratio, gender selection, reproductive technologies

## Abstract

**Simple Summary:**

The rabbit production industry would greatly benefit from a technology that allows producers to obtain a more predominantly male or female offspring, accordingly to the goals of each production farm. Our review highlights the current and future developments in rabbit sperm sexing technologies, as well as the potential impact of implementing these methods in cuniculture. Given the remarkable technological progress made in recent years, there remains a pressing need for further research aimed at developing a cutting-edge technology that not only exhibits high efficiency but also offers cost-effectiveness, thus enabling its widespread adoption across the cuniculture industry.

**Abstract:**

Sperm sex selection is a longstanding challenge in the field of animal reproduction. The cuniculture industry, in particular producers of males or females for breeding purposes, would greatly benefit from the pre-selection of the offspring’s sex. This review article overviews the current and future developments in rabbit sperm sexing technologies, as well as the implications of implementing these methodologies in cuniculture. The first attempts of sperm sexing were performed in rabbits; however, a both efficient and cost-effective methodology was not yet developed for this species. Those included sperm sexing according to differences in sperm density, surface electric charge, pH susceptibility, antisera reaction, and flow cytometry. Separation by flow cytometry has proven to be efficient in rabbits, yielding fractions with approximately 81% and 86% purity for X- and Y-sperm, respectively. However, it is not cost-effective for cuniculture and decreases sperm quality. The advantages, limitations, and practical considerations of each method are presented, highlighting their applicability and efficiency. Furthermore, herein we explore the potential of immunological-based techniques that overcome some of the limitations of earlier methods, as well as recent advancements in sperm sexing technologies in other animal models, which could be applied to rabbits. Finally, the challenges associated with the development and widespread implementation of rabbit sperm sexing technologies are addressed. By understanding the advantages and limitations of existing and emerging methods, researchers can direct their efforts towards the most promising directions, ultimately contributing to a more efficient, profitable, and sustainable cuniculture.

## 1. Introduction

The cuniculture industry has a considerable economic impact on the world. A total of 893,631 tonnes of rabbit meat were produced in 2020, according to the Food and Agriculture Organization of the United Nations, with China solidifying its position as the world’s top producer, accounting for a staggering 456,600 tonnes of the total production [1,2]. In 2021, the production of rabbit meat resulted in a total export value of 123 million € and an import value of more than 114 million € [2]. Spain emerged as the largest exporter of rabbit meat, with a total export value of approximately 29 million €, while Germany was the largest importer with an import value of 30 million € [3].

Modern cuniculture divides into breeding and production farms. Breeding farms raise the male (buck) or female (doe) progenitor rabbits, which will breed the producing animals; in turn, production farms raise rabbits for slaughtering and meat consumption. Does are more valuable in the breeding sector, whereas bucks are preferable for meat production [4]. Until now the decision to use bucks or does for meat production was based on factors such as growth rate, feed efficiency, and breeding performance [5]. However, the meat quality, namely aroma flavor characteristics, starts to carry significant weight in consumers’ purchase decisions, which may influence the choices made by the food industry [6,7]. Although there is no consensus in the literature on the differences in the quality of female and male rabbit meat (reviewed in [8]), it is already reported that some people prefer female rabbit meat over male meat due to the latter’s stronger odor, which can be possibly explained by the distinct profile of volatile odorants [9]. Given this, it is possible that a preference for female meat by the food industry may emerge in the future to meet consumers’ preferences and remain competitive in the market. Moreover, companies specializing in the sale of breeding does have a greater incentive to retain female animals in their production, which in the North-West of Spain can be worth 6 pounds more than males (approximately, 7.5 € in 2008), while buck-producing farms or meat production farms, for instance, have a greater incentive to retain male animals [4].

This leads to a surplus of rabbits of the unwanted sex in animal production, which raises concerns about: animal welfare, since less valuable rabbits may be subjected to worse care conditions and early culling [10]; environmental impact, due to manure and feed production for animals of inferior economic value [11]; and reduced profitability, since the surplus animals still require feeding, housing, and veterinary care, whose costs may exceed their potential value [12].

To mitigate these implications, the cuniculture industry would benefit from sperm sexing technologies that enable the pre-selection of the offspring’s sex according to the goals of each farm. Although the first attempts at sexing spermatozoa were performed in rabbits, a both efficient and cost-effective methodology was not yet developed for this species, unlike other animal production industries such as cattle, which already benefit from sexed semen at a reasonable cost for beef and dairy production [13,14,15,16].

This review aims to provide a comprehensive analysis of the methodologies underlining the attempts for sexing rabbit spermatozoa and the latest advancements in sperm sexing technologies, as well as the practical implications and perspectives of implementing sperm sexing in cuniculture.

To conduct the literature review, an extensive search was performed in PubMed, Scopus, Web of Science, and Google Scholar to gather papers, as well as conference ab-stracts, available online until 31 March 2023, related to the topic of interest. The search terms included “rabbit” and/or “sperm sexing”, “sperm sorting”, “sex selection”, or “sexed semen”. All research papers that documented attempts of sperm sexing using rabbit sperm ejaculates were included. Additionally, papers describing sperm sexing in other species were considered if they had historical significance in the field of sperm sexing or the potential to contribute to advancements in rabbit sperm sexing. Furthermore, to provide a more critical evaluation of each discussed method, additional works available online were gathered. The search conducted until 14 July 2023, using the same databases mentioned earlier, included the use of other relevant terms such as “sperm” or “spermatozoa”, as well as terms like “pH susceptibility”, “density”, “sedimentation”, “electric charge”, “electrophoretic separation”, “antisera”, “DNA content”, “laser ablated”, or “dead sperm”. Works entirely published in languages other than English were excluded.

## 2. Attempts for Sexing Rabbit Spermatozoa

The earliest attempts for sexing rabbit sperm, prior to or unrelated to the flow cytometry breakthrough, were largely unsuccessful due to a lack of reproducibility, accuracy, and consensus between authors. However, they should not be overlooked, since they began to unveil differences between X-chromosome-bearing sperm (X-sperm) and Y-chromosome-bearing sperm (Y-sperm) that later were crucial for the development of the most recent and effective methodologies [17,18,19,20]. The most representative papers of rabbit sperm sexing are listed in Table 1 and the underlining methodologies will be further described in this section.

### 2.1. Sperm Sexing through Density Gradients

The first attempts at sperm sexing were based on the differential densities of X- and Y-sperm. The X-chromosome is heavier than the Y-chromosome since it accommodates more genes. In rabbits, the X-sperm was shown to have 3.9% more DNA than the Y-sperm [43]. Thereby, some authors had the theory that X- and Y-sperm sediment at different rates and at least two distinct fractions should be obtained, after centrifugation of a semen sample, corresponding to a majority of either X- or Y-sperm [17,20,24,44,45].

Although some authors advocate that this difference in the DNA content does not significantly influence the density of the spermatozoa, others reported successful separation [17,46]. In 1962, Bhattacharya stated that the sex of the rabbits born after the sedimentation of semen was related to the sedimentation rate, with the upper fractions being mostly responsible for male offspring (around 77.4%) [22,47]. Other authors documented that the obtained fractions do not significantly relate to the sex chromosome of the spermatozoa [21,23,24,25,48,49]. For example, Lush tried to separate rabbit X- and Y-sperm using an ordinary centrifuge [21]. However, his experiments were unsuccessful due to experimental limitations, which motivated other authors to try and improve the procedure [21,49]. Thus, Bedford and Bibeau, and Beatty also attempted to sort rabbit spermatozoa by sedimentation, while employing similar methodologies [23,24]. Altogether, they agreed that sedimentation over density gradients is not a viable sexing method [21,23,24].

Other authors tried more inventive approaches that resulted in a more imbalanced sex ratio in rabbit semen but were not sufficient to accurately control the sex of the offspring. Stambaugh and Buckley obtained predominantly male offspring following the insemination of rabbits with spermatozoa from the supernatant of discontinuous dextran density gradients (4–24%) [25]. Zavos and Quinlivan attempted to sex rabbit and human spermatozoa with albumin gradients, respectively, but did not achieve significant or consistent results among samples [26,50]. Further, Copello and Hussein’s experiments resulted in a higher proportion of female offspring when rabbit semen was separated by Percoll gradient, whereas sexing by swim-up procedures attained an increased number of males [27,28]. While attempting to separate human X- and Y-sperm using Percoll gradients, Kaneko and collaborators observed a decrease in Y-sperm and an increase in X-sperm with increasing density [51]. Also, Hedge used Ficoll-Metrozoate gradients but to enrich human samples in Y-spermatozoa, managing to achieve an accuracy rate of approximately 63% [52].

The major limitation of the aforementioned methods was their low accuracy and lack of reproducibility, since similar experiments achieved divergent results, although sperm sexing through density gradients would be a low-cost methodology for sperm sexing [17,49]. Branham advocates that the general failure in separating X- and Y-sperm through density gradients is the lack of information about the factors that influence sperm sedimentation, such as the composition of the medium [53]. He then advised that, to separate the two fractions, one had to control flocculation, and demonstrated that suppressing this phenomenon had an impact on the results [53]. Nonetheless, the highest accuracy ever reported has not exceeded 75%, which prevents the widespread application of this method of sperm sexing.

It is also worth noting that normal and abnormal spermatozoa have different densities, with mature morphologically normal spermatozoa having a slightly higher density than immature and morphologically abnormal spermatozoa (reviewed by [54]). Previous research has shown that it is possible to separate human ejaculated spermatozoa into four fractions representing different stages of maturation by density gradient [54]. Moreover, although it can be affected by the gradients used, it also seems possible to separate spermatozoa with different levels of DNA integrity using density gradients [55]. Therefore, among others, the presence of both fully mature and immature spermatozoa and variations in spermatozoa DNA integrity in ejaculates may affect the ability to accurately separate X- and Y-sperm based on low-density variations that may exist due to the sexual chromosomes they bear.

### 2.2. Sperm Sexing through Electrophoresis Based on Surface Electric Charge Differences

Spermatozoa present a negatively charged surface due to the sialic acid secreted by the epididymal epithelium [56]. It has been described that due to the different amounts of sialic acid content exposed, X- and Y-sperm exhibit differently electrically charged plasma membranes. In particular, the zeta potential of human spermatozoa is −16 mV and −20 mV for Y- and X- sperm, respectively; therefore, separation by electrophoresis was also tested in rabbit semen [30,46,57].

In the 20th century, some authors stated that when placed in an electric field, two roughly same-sized populations of rabbit spermatozoa migrated toward the anode and cathode [30,31,32]. After the insemination of rabbits with either fraction, there were reasons to believe that those fractions were mostly composed of X- or Y-sperm, respectively. However, data from different studies were not consistent and similar experiments achieved contradictory results [30,33,46].

As early as 1933, Koltzoff & Schröder reported a successful separation of rabbit X- and Y-sperm through electrophoresis. The anode-migrating fraction produced predominantly female offspring after insemination, whereas the cathode-migrating fraction originated more male progeny [29]. Later, Gordon reported similar results, but others were unable to do so [32]. Sevinç performed four similar experiments with a few changes in the technique, namely different apparatus, buffers, sperm concentrations, and electric power, and achieved inconclusive results [33]. Bangham and Nevo were also unable to corroborate Schröder and Gordon’s results [30,31].

These authors argue that more variables are at play when considering spermatozoa migration in an electric field. For instance, the exposed sialic acid content could depend on other aspects of the sperm cells, such as cell quality and viability [17,30,31,33]. In fact, in 2011, Ainsworth et al. concluded that electrophoretic isolation of human spermatozoa was independent of genotype but instead relied upon the sperm surface glycoproteins. Furthermore, the separation of spermatozoa appeared to be associated with quality, which was associated with high levels of surface sialic acid [58]. These findings seem to help explain the inconclusive results obtained in the first attempts at sexing rabbit spermatozoa through electrophoresis and why it seems unfeasible.

### 2.3. Sperm Sexing Based on pH Susceptibility

Based on the claim that X- and Y-sperm are differently affected by pH, several authors tried to control the offspring sex ratio by changing the pH of either the seminal plasma or the female vagina [36,59]. In 1932, Unterberger stated that an alkaline seminal plasma favors male offspring, while a very acidic vaginal environment favors female progeny ([34] as cited by [36]) Later, Wakim also noted that vaginal pH affected the sex of rabbit progeny, with lower pHs (6.55 to 7.34) originating more female offspring and higher pHs (above 7.55) producing more males [35]. Muehleis sought to explain Wakim’s experiments but ultimately demonstrated that altering the pH of the seminal fluid did not significantly affect the sex ratio, suggesting that the pH does not directly affect the sex of the progeny. Hence, the mechanism through which altering intravaginal pH influences the sex of the offspring appears to be more complex than initially anticipated, rendering this method of separation unreliable [36].

In 2021, Park and collaborators also tested the influence of the pH on the sex ratio deviation using boar semen samples. They concluded that sows inseminated with spermatozoa stored for 1 day at a pH of 6.2 gave birth to 1.5 times more females than sows inseminated with spermatozoa stored at a pH of 7.2. Nevertheless, for sows inseminated on day 2, only a tendency was observed [60]. More recent findings have demonstrated that other factors per se, such as temperature and incubation period, may impact the X:Y chromosome ratio of human live spermatozoa, while the influence of different pH conditions (6.5–8.5) was not so linear [61]. Furthermore, it is described that when subjected to identical stress conditions, Y-sperm exhibited higher expression of apoptotic proteins and lower survival over time compared to X-sperm [61]. This indicates that, under certain conditions, Y-sperm may have a lower probability of successful fertilization compared to X-sperm, influenced by multiple factors beyond just pH, which may also explain the inconsistency between studies.

### 2.4. Sperm Sexing through Antisera Reaction

In theory, rabbit sperm could be sorted by creating antisera that selectively react with either X- or Y-sperm, leading to the destruction of one type and allowing for the recovery of the other, with a high degree of purity [38,62]. Burkov proposed the use of bird sperm as an immunizing antigen against either X- or Y-mammalian sperm [37]. The offspring of rabbits intra-vaginally immunized with bird sperm before insemination was more predominantly female (58%), probably due to an immune attack on Y-sperm [37]. Hancock also tested anti-cock sperm sera in rabbit semen and evaluated their effect on the sex ratio of offspring, observing a more predominantly female offspring (58%), when compared to more males born after incubating rabbit sperm with normal sera (64%) [38]. Although promising, both his and Burkov’s investigations were insufficient to declare this methodology accurate, since it did not significantly deviate from the normal 50:50 sex ratio [37,38].

The male-specific H-Y antigen has long been investigated regarding its potential at discriminating X- and Y-sperm. However, most of the attempts at sexing rabbit semen failed, and authors disagree on the differential expression of H-Y in X- and Y-sperm [17,18]. In 1983, Zavos demonstrated that intra-vaginal administration of H-Y antisera in rabbits before insemination resulted in significantly more females in offspring (74.2%), suggesting that this practice is a valuable simple method to ensure a more predominantly female progeny [39].

Nevertheless, other authors’ research suggested that the anti-HY does not bind specifically to Y-sperm. Prasad performed sexing experiments with bovine samples and concluded that H-Y was present on both X- and Y-sperm cell surfaces [63]. Sills and colleagues also found that the anti-HY antibody binds to X- sperm as well. Despite detecting a slight discrepancy in the expression of this antigen between X- and Y- sperm, they considered it insufficient for sperm sexing to be reliable [64]. Hence, although initially it was thought that only Y-sperm expressed H-Y antigen, this was later contradicted. Since there is not sufficient accurate evidence that H-Y was differently expressed between the two types of sperm, this approach is not recommended for sperm sexing [18,63,64].

### 2.5. Other Approaches for Sperm Sexing

Some authors attempted to control the sex of the progeny through less rigorous methodologies, which resulted in discrepant results. Hays observed that a higher frequency of sexual activity in male rabbits was associated with a higher proportion of female offspring, while not being able to explain that phenomenon [40]. Later, D’Amato et al. performed a similar experiment but did not find a significant correlation between the sex of the offspring and the frequency of ejaculation or sexual activity of male rabbits [41]. Therefore, he discouraged the use of this method to control the sex ratio in rabbits [41].

### 2.6. Sperm Sexing through Flow Cytometry

Despite numerous attempts to separate X- and Y-sperm, the efficacy of these methods in manipulating the sex ratio in rabbits has been unconvincing. It was with the introduction of flow cytometry that the first promising and repeatable results appeared, and it did not take long for the technology to be adapted for use in other species [19].

Some of the first studies that recognized fundamental differences in the DNA content of the sex chromosomes, which translate into differences in the DNA contents of X- and Y-sperm, date back to 1968. Those observations were performed by different techniques, such as ultraviolet microspectrophotometry (as cited by [43]) and fluorescent and Feulgen techniques (as cited by [65,66]). In 1976, Gledhill and collaborators attempted to measure spermatozoa DNA by flow cytometry, seeing the potential of these differences not only for quality control in enrichment procedures but also as a foundation for a sperm sexing technique (reviewed in [43,67]). Flow cytometry utilizes fluorochromes specific for DNA to yield a highly accurate method for determining relative DNA content in thousands of cells per second. As a result of the disparities in DNA content between X- and Y-chromosomes, two distinguishable cell populations can be recognized [43]. Subsequently, numerous studies emerged documenting the distinctive DNA variations between X- and Y-sperm in multiple species, namely rabbit, bull, ram, boar, and mouse [43,68].

In 1983, Keeler and collaborators successfully sorted viable spermatozoa with a bimodal fluorescence profile using a commercially available fluorescence-activated cell sorter [69]. In 1986, Johnson successfully sexed spermatozoa based on the DNA differences, using a flow cytometer cell sorter (unpublished data), and in 1988 also Morrell and co-workers sexed mammalian sperm using flow cytometry and inseminated females with both fractions, observing a tendency towards sex ratio deviation, despite it not being statistically significant [42,70,71]. However, it was in 1989, after optimization to maintain sperm viability, that the first results were released describing the sexing of spermatozoa from rabbit samples [42,70]. Both fractions were sorted simultaneously at a rate of approximately 80–90 intact X-sperm and 80–90 intact Y-sperm per second. Contrary to most previously proposed methods for altering offspring sex ratio, this one proved to promote a consistent X- or Y-sperm enrichment. The X-sorted sperm had a purity of 86% and the Y-sorted sperm had a purity of 81%. The does were later inseminated with both fractions to evaluate the accuracy in vivo, showing a relatively reduced kindling rate of 28%. Nevertheless, almost all the offspring born from does inseminated with X-sperm were females (94%) and most of the offspring born from does inseminated with Y-sperm were males (81%). This study made significant progress in sex preselection for mammals, but they were still facing challenges that prevented widespread use of the method. These included a limited sorting capacity (3.5 × 10^5^ spermatozoa per hour); higher embryo mortality, that they thought might be related to the use of DNA fluorochrome or manipulation of the uterus during surgical insemination; and high equipment costs [42]. Not only were more studies needed to improve the sorting speed, but also more studies to determine the minimum number of sperm per insemination dose required to achieve acceptable pregnancy rates. Nonetheless, pioneering works like this one laid the foundation for the development, improvement, and widespread successful use of flow cytometry in sperm sexing, namely in swine, horses, sheep, goats, dogs, cats, deer, elk, dolphins, and water buffalo, and commercially in dairy and beef cattle industries, marking a significant milestone in the history of reproductive science [72,73,74,75]. This technique was first patented in 1992, and several improvements have been patented since, including an improved orienting nozzle and a high-resolution flow cytometer, contributing to better speed and purity of sperm sorting [72,76,77,78,79].

Other significant advancements have been implemented over time, with enterprises now offering sexed semen products that lead to up to 8 to 14% higher conception rates compared to sexed semen obtained by earlier technologies [80,81]. Various options, such as SexedULTRA 2.1M, SexedULTRA 4M corresponding to a higher semen dose of 4 million spermatozoa per straw, and UltraPlus, which is described as the highest fertility gender-sorted semen with 3% higher conception rates than SexedULTRA 4M, are mainly available for application in cattle [80]. When females express estrus, SexedULTRA semen products lead to similar pregnancy rates obtained with unsexed semen [81,82]. Other combinations are available, such as UltraPlus High Purity, which offers 96–97% gender accuracy [80].

In addition to cattle, advances in extenders and technologies, like SexedULTRA, have also led to the development of caprine sexed semen since 2015, as reported by González-Marín et al. [83]. On the other hand, even today it is still not profitable to use semen sexed by flow cytometry in cuniculture [4].

## 3. Sperm Sexing through Immunological Methods: A Promising Weapon

As mentioned, profitable rabbit sperm sexing through flow cytometry was not yet achieved, particularly due to the high cost of the equipment and inherent sperm doses, and sperm quality decrease [18]. In 1992, Johnson and collaborators already pointed out that the most reasonable and economical way to obtain sexed semen might be through the use of a surface membrane marker specific to each sex, with investigations ongoing at the time to find such a marker in bovine samples [70,84]. Other authors share the same opinion [18]. Thus, an immunological method applied to rabbit sperm might greatly benefit the cuniculture industry.

As previously described in this review, some immunological approaches were already tested in rabbit sperm samples, such as the use of antisera to selectively react with either X- or Y-sperm, but were not consistent enough in terms of success and/or did not promote a great sex-ratio deviation. According to Braun et al. (1989) and Jasin and Zalamea (1992), since spermatogenic cells are connected by intercellular bridges until the end of spermatogenesis, each cell containing an X or Y chromosome matures at the same time, which can translate into a similar origin, maturation, and functions of the X- and Y-sperm. Yet, it does not occur a total share of the gene products (reviewed in [85]). Also, it is described that proteins expressed in post-meiotic germ cells, namely spermatids, are crucial for spermatogenesis and differentiation of X- and Y-sperm, while most sex chromosome genes are not translated in later spermatogenesis stages (reviewed in [86]). Nonetheless, if there are genomic DNA differences between X- and Y-sperm, then there may be a differential expression of genes and, as such, molecular differences regarding proteins of X- and Y-sperm, as proved by recent genomic and proteomic studies [18,85]. The quest for a distinct marker of one of the spermatozoa types may encounter certain obstacles, namely the inferior number of genes encoded by the Y chromosome when compared to the X chromosome (reviewed in [85]). Once a protein specific to the rabbit X- or Y-sperm is identified, sperm sexing can be achieved by coating magnetic beads with a directed antibody, followed by incubation of the semen sample with the coated beads. The X- or Y-sperm cells would then bind to the beads and remain retained in the tube upon application of a magnetic field, allowing for the recovery of the opposite pool of spermatozoa [87]. This could be a valuable approach that combines innovation, cost-effectiveness, and scalability, making it an option for revolutionizing rabbit breeding practices.

Sperm sexing by immunological approaches can also work based on protein function. For example, according to Umehara and collaborators, the activation of TLR7/8, encoded by genes on the X chromosome in species such as mice and cattle, leads to changes in ATP production, which promotes a reduction of motility in X-sperm as compared to Y-sperm. This immunological-related modification opens up the possibility of sexing sperm based on their altered motility [14,88]. However, in European rabbits, TLR8 is linked to chromosome 13 and TLR7 is absent [89].

Currently, although not for rabbits, immunological-based sperm sexing kits are already available in the market for some mammalian species, which stands as evidence for the broad versatility and practical applicability of immunological methods compared to flow cytometry. Nuri Science Inc. launched two sperm sexing kits for bovine, equine, caprine, ovine, canine, and porcine sperm, whose commercial names are WholeMom and WholeMan [90]. WholeMom is based on the agglutination of the Y-sperm, which favors fertilization by the X-sperm. WholeMan, in turn, increases the motility of the Y-sperm, promoting male gestations, and is only available for cattle [90]. Both kits are easy to apply before artificial insemination and the accuracy ranges from 70–90% depending on the species [90,91]. There is limited literature available to substantiate the results disseminated by the company. Nonetheless, according to a paper published in 2018, where cow oocytes were in vitro fertilized with frozen-thawed semen samples, treated or not with the WholeMom kit, the female ratio was significantly higher in the treated group (85.4%) compared to the control group (47.2%). Conversely, the treatment had a significant impact on the fertilization rate, with a lower rate observed in the treated group (66.9%) compared to the control group (75.0%) [91].

EMLAB Genetics also delivers sperm sexing kits for either female or male gestations, with accuracy between 65 and 90% depending on species and sperm sample condition (fresh and frozen-thawed), by enhancing the fertility and motility of the X- or Y-sperm [15]. The kit is also easy to implement and is available for bovine, equine, caprine, ovine, canine, and porcine sperm [15].

Although several studies have long focused on identifying the spermatozoa proteome, namely the differential proteome profiles of X- and Y-sperm (reviewed in [85,87,92]), it was not until February 2019 that the first study on the identification and quantification of rabbit sperm proteins was published [93]. Therefore, sperm sexing in rabbits by an immunological approach has still a long way to go. It may prove beneficial to conduct a comprehensive proteomic analysis on X and Y sperm cells sourced from diverse animal species, thereby enabling the identification of potential shared targets that could be adapted for use in rabbits [85].

## 4. Sperm Sexing Methods in Development

More ambitious methodologies are being developed in other animal models, capable of being translated into other animals, such as rabbits. Douglas and colleagues have been working on the development of CRISPR-Cas9 genetic tools to produce male- or female-only litters with 100% efficiency in mice. The authors state that this technology is a step forward to create an ethical and economical sexing methodology since it is the first attempt at sperm sexing with CRISPR-Cas9 in mammals [94,95]. Previously, a similar methodology was applied by Gamez et al. to develop a *Drosophila melanogaster* Y-linked transgenic line that could be used for instance for sex-related pest control, as well as serve as a basis for future Y-gene editing [96]. This was also attempted in silkworms [97], mosquitoes [98] and zebrafish [99].

Furthermore, Dominguez and colleagues separated donkey X- and Y-sperm with magnetic nanoparticles, acquiring the X-sperm fraction with a 90% accuracy. This was achieved due to the different X- and Y- Z potential that causes sperm to migrate distinctively along an electrophoretic field [100]. This is an advantage compared to flow cytometry, since sperm quality parameters, such as viability and motility, were unaffected. Moreover, this technique is easier to perform and less expensive [100].

Currently, the ABS Global company commercializes a gender ablation kit called Sexcel sexed genetics^TM^; it sorts X- and Y-sperm with around 85% accuracy by staining the DNA with Hoechst 33342. Subsequently, a laser destroys the Y-sperm, and the X-sperm portion is saved [16,101,102]. This technology seems advantageous when compared to flow cytometry: it provokes less cell damage since cells are not divided into droplets and an electric field is not required. Moreover, the company advertises that with this sexed semen, a 90% relative conception rate to unsexed semen can be achieved [16]. While the presence of non-viable spermatozoa can potentially impact the remaining ones, a study by Faust et al. demonstrated that the bisected spermatozoa and their debris present in cattle semen sexed through laser-based cell destruction did not impact conception rate [103]. Furthermore, it has been described that samples sexed using this method yield comparable results to other available methods for cattle [102]. Nonetheless, Perry et al. advise caution when employing gender-ablated semen in animals that do not exhibit any signs of estrus since the pregnancy rates were significantly lower compared to conventional semen, while in animals experiencing estrus or with partially activated patches, gender-ablated semen achieved pregnancy rates ranging from 82% to 89% of those achieved with conventional semen [102].

Additionally, De Luca et al. introduced Raman spectroscopy as an accurate (90% on average), highly efficient, non-invasive, and non-destructive tool for bovine sperm sexing [104]. Later, Ferrara et al. combined Raman spectroscopy with digital holography to characterize spermatozoa’s morphological and biochemical characteristics more efficiently, as well as to identify X- and Y-sperm [105].

While it is not a direct sperm sexing method, studies have suggested that the maternal diet could influence the sex ratio of the offspring in rodents, ruminants, and primates (reviewed in [106]). However, some authors have argued that the maternal body condition rather than diet might be influencing those results; there are few controlled studies on the topic to draw reliable conclusions [106,107]. Recently, Alhimaidi et al. showed that more sheep males were born of mothers under a high-sodium, -potassium, and -chloride diet (77.27%), whereas more females were born from mothers under a high-calcium and -magnesium diet (72.72%), both groups without significant changes in body weight [108]. Despite the controversy, this suggests that maternal diet control that does not affect the body condition could be promising as a methodology for modifying the sex ratio of offspring.

Sperm sexing methods continue to be the fastest-growing technologies in the animal production and artificial insemination sector. The demand for sexed sperm persists despite the available technologies, particularly to find novel solutions that are cheaper and have minor negative impacts on the final sexed sample quality [19,101]. To provide a comprehensive overview of the potential of each discussed technique for rabbit sperm sexing, a qualitative comparison of cost, scalability, and their potential impact on production efficiency has been systematically organized and presented in Table 2.

## 5. Conclusions and Future Perspectives

In 2008, Garner and Seidel stated that “The most sought-after reproductive biotechnology of all time, selection of sex at conception, has a long history of great optimism, along with many disappointments” [76]. It is noteworthy that after 15 years this remains true, especially regarding rabbit sperm sexing.

After examining the various technologies tested on rabbits, it has become evident that a considerable number of them are not reproducible or do not provide accurate sexing [33,36,39]. Even the ones that allow for satisfactory accuracies, such as flow cytometry, result in a significant decline in semen quality, leading to impaired fertilization rates, and expensive insemination doses, which makes their application financially unfeasible in cuniculture [4]. Notwithstanding, immunological methods show promise and have the potential to become a viable alternative in rabbit farming [18].

Despite the potential of immunological methods, several challenges need to be overcome. Obtaining sufficiently pure fractions of rabbit X-sperm and Y-sperm for proteomic studies is challenging due to the limited options for sexing, therefore compromising the detection of differences at a proteome level. One of the major hurdles is also the lack of a complete proteome associated with the rabbit Y chromosome in UniProt [116]. Moreover, as mentioned in the review, the Y chromosome has fewer associated genes compared to the X chromosome, further reducing the chances of finding a specific target for the Y-sperm, although potential targets may be associated with autosomes too. Additionally, many of the commercially available antibodies have not been tested or developed for use in rabbits, which limits their effectiveness and biomarkers validation; in fact, rabbits are frequently the antibodies’ host, which further limits their subsequent use in samples from this species [117].

Other challenges arise from the implementation of the technique. Certain immunological methods that rely on the binding of an antibody to a specific protein of one type of sperm, allowing it to couple to a magnetic bead or to be retained on a matrix, require the target protein to be located on the plasma membrane and have an accessible transmembrane domain [18]. However, not only are membrane proteins inherently challenging to detect using mass spectrometry, but many commercially available antibodies lack an immunogen directed towards the transmembrane domain, making it difficult to find one suitable for use in such methods [118,119]. After determining a protein target, the only solution may indeed involve de novo design and development of an antibody. Moreover, compared to other livestock species, such as bulls, rabbits have a lower economic value. Consequently, this imposes a constraint on pricing for sexed semen doses intended for use in artificial insemination, which should remain at relatively low values. Therefore, the cost of the sexing technology must remain within reasonable limits to ensure its broad applicability in cuniculture.

Conversely, rabbits possess traits that can potentially facilitate the cost-effective implementation of sperm sexing techniques of slightly inferior accuracy. The use of techniques of low accuracy is not profitable for application in species that typically give birth to only one offspring at a time. Nevertheless, rabbits are polytocous animals, so they give birth to more than one offspring at a time [4]. For example, New Zealand rabbits, widely used for meat production, have an average litter size of 7–8 kits [120]. Therefore, smaller but consistent deviations in the sex ratio of the offspring could still be advantageous for farmers since it will occur in a significant number of inseminated does. Moreover, compared to monotypic species, such as cattle, the use of sexed semen with an inferior accuracy in rabbits would allow for a proportionally higher chance of enrichment since several oocytes can be fertilized by spermatozoa [4]. When using sexed semen doses with an accuracy of 70%, 80%, or 90%, the increase in rabbits of the desired sex per litter will be approximately 40%, 60%, or 80%, respectively. This increase is achieved while considering a similar fertilization rate and litter size as when using unsexed semen. The point in the production chain where this technology will yield the highest unit economic gain is in the production of grandparent and parent female breeding lines. The males resulting from the same litters as the breeding females have a lower feed conversion rate than rabbits from litters suitable for meat production [121]. According to the Madrid/Loncun stock exchange, the price of live rabbits for meat (2.2–2.5 Kg) has fluctuated between 2.40 € and 2.50 € since the beginning of 2023, with an average price of 2.42 € [122]. As a result, a producer who chooses to fatten and sell these males for meat will make little to no profit, given the production costs. On the other hand, grandparent and parental females can be sold at prices significantly higher than their male counterparts. Therefore, the implementation of a rabbit sperm sexing technique would enable targeted breeding programs, faster genetic gain, and a more effective response to market demands, resulting in increased export capacity and enhanced profitability for companies.

Additional research that combines genomic and proteomic analyses across various animal species is necessary to identify sex-specific proteins that can be used to improve animal production outcomes. These efforts will contribute to the development of effective and less invasive sexing technologies. Additionally, they may lead to the discovery of targets specific to rabbits, ultimately contributing to the improvement of cuniculture and production practices, alongside other breeding strategies [123].

## Figures and Tables

**Table 1:** Rabbit sperm sexing attempts categorized by sexing principle. Categorization of studies on rabbit sperm sexing based on the sexing principle employed, including accuracy of each method, the targeted chromosome, and authors and publication years.

Sexing Principle	Methodology	Accuracy	X or Y	References
Density	Differential sedimentation in an ordinary centrifuge	No effect	X and Y	[21]
	Sedimentation in a colloidal medium (egg yolk and glycocoll solution) of a particular viscosity and density	More males in upper fractions (77%) than lower (28%)	X and Y	[22]
	Sedimentation with a customized medium	No effect	X and Y	[23]
	Separation accordingly to buoyant density of the spermatozoa	No effect	X and Y	[24]
	Discontinuous dextran density gradients (4–24%)	68% males	Y	[25]
	Albumin gradients	No effect	Y	[26]
	11 discontinuous Percoll gradients	No effect	X	[4]
	Percoll gradient and swim-up	Percoll gradient: 68 ± 2% females; swim-up: 64 ± 2% males	X and Y	[27]
	Percoll gradient and swim-up	Percoll gradient: 66% females; swim-up: 75% males	X and Y	[28]
Surface electric charge	Electrophoretic separation in a Michaelis apparatus	100% females (anode); 80% males (cathode); 50% males and 50% females (central fraction)	X and Y	[29]
	Electrophoretic separation in different conditions	No effect	X and Y	[30]
	Electrophoretic separation in different conditions	No effect	X and Y	[31]
	Electrophoretic separation at pH 7.1	71% females (anode); 64% males (cathode)	X and Y	[32]
	Electrophoresis of spermatozoa using V-shaped electrophoretic cells having agar gel stoppers between each electrode and its related lateral chamber. The three-chambered cell was used in the first three experiments, and the seven-chambered cell was used in the fourth experiment.	Inconclusive	X and Y	[33]
pH susceptibility	Control of the pH of the seminal plasma or the vagina	Higher seminal plasma pH: more male offspring; lower vaginal pH: more female progeny	X and Y	[34]
	Control of the vaginal pH	Lower pH (6.55–7.34): more female offspring; higher pH (>7.55): more males	X and Y	[35]
	Altering the seminal fluid pH	No effect	X and Y	[36]
Antisera reaction	Antisera (cock sperm)	58%	X	[37]
	Antisera: anti-cock sperm sera incubated with rabbit semen	58%	X	[38]
	Intra-vaginal administration of H-Y antisera	74%	X	[39]
Other approaches	Increased sexual activity	78%	X	[40]
	Increased frequency of ejaculation	No effect	X	[41]
Flow cytometry	Rabbit semen sorted simultaneously at a rate of approximately 80–90 intact X-sperm and 80–90 intact Y-sperm per second by a modified EPICS V flow cytometer/cell sorter, based on DNA content of X- and Y-sperm	Purity of 86% for X-sperm (94% in vivo) and 81% for Y-sperm (81% in vivo)	X and Y	[42]

**Table 2:** Comparison of the sperm sexing methods overviewed in the review. Provided are summaries of cost, production scalability, and potential impact on production efficiency.

Sexing Principle	Cost	Scalability	Production Efficiency
Sperm sexing through density gradients	Relatively low cost as it involves simple laboratory equipment and reagents [17,21,24,47]	Can be scaled up easily [27,49,55]	Low to moderate impact on production efficiency due to the need for additional processing steps. However, its possible effectiveness and accuracy depend on several factors. The lack of consistency may further impact the overall production efficiency [21,25,27,28,48,49,51,55]
Sperm sexing through electrophoresis based on surface electric charge differences	Moderate cost due to the specialized equipment required for electrophoresis [33,58,109]	May be challenging to scale up [58,110]	Low to moderate impact on production efficiency. The results seem to be influenced by different experimental conditions and variables, such as sperm quality, viability, and surface glycoproteins, potentially affecting the efficiency and reliability of the method [29,32,33,46]
Sperm sexing based on pH susceptibility	Relatively low cost as it involves simple laboratory equipment and reagents [35,36,61]	Can be scaled up easily [60,61]	Low impact on production efficiency. However, the relation between pH and sex ratio is not clear and seems to be influenced by other factors, which makes the method unreliable [34,35,60,111]
Sperm sexing through antisera reaction	Moderate to high cost due to the need for specific antisera and specialized reagents [37,39]	May be challenging to scale up [38,63]	Low impact on production efficiency. There is not sufficient accurate evidence that H-Y is differently expressed between X- and Y-sperm. It may have limitations in terms of accuracy and speed [18,37,38,64]
Sperm sexing through flow cytometry	Relatively high cost due to the need for specialized flow cytometry equipment and expertise [18,75,76]	Can be scaled up, but costs and technical expertise may limit implementation, depending on resources [4,42,72,73]	High impact on production efficiency as it allows rapid and accurate sex sorting of sperm cells. However, it requires skilled operators and sophisticated equipment [69,71,112]
Sperm sexing through immunological methods	Moderate to high cost depending on the specific method and reagents used [15,76,90]	May be challenging to scale up [88]	Moderate to high impact on production efficiency. It requires additional processing steps and may have limitations in terms of speed and accuracy depending on the target [15,88,91]
Sperm sexing through CRISPR-Cas9 genetic tools	Relatively high cost due to the need for gene editing reagents and equipment [96,97]	Can be challenging to scale up [95,96]	High potential for production efficiency if optimized properly. It offers precise genetic manipulation, but further development and validation are needed for commercial applications [94,95,96]
Sperm sexing with magnetic nanoparticles	Moderate to high cost due to the need for specialized nanoparticles and equipment [100]	Can be scaled up, but may require optimization for large-scale implementation [113,114]	Moderate to high impact on production efficiency. It enables efficient separation of sperm cells but requires additional steps and optimization for different species [100,114]
Sperm sexing with Raman spectroscopy combined or not with digital holography	Relatively high cost as it requires advanced spectroscopy and imaging equipment [104]	Can be challenging to scale up [105]	High impact on production efficiency as it offers label-free and non-invasive analysis. However, it requires further development and validation [104,105,115]
Sexing through the feed of mothers	Varies depending on the nutritional requirements and supplements needed [107,108]	Can be scaled up easily. However, it may require careful consideration of feed formulation and management practices [106]	Variable impact on production efficiency and reliability depending on the species, nutritional adjustment used, and the desired sex ratio. It may be challenging, not providing the same level of precision as direct sperm sexing methods [106]

## Data Availability

The datasets generated during and/or analyzed during the current study are available upon request from the corresponding authors.

## References

[1] United Nations Data Retrieval System|Food and Agriculture Organization of the United Nations (FAO) Data Data Series, Meat. http://data.un.org/Search.aspx?q=meat+datamart%5BFAO%5D.

[2] TRIDGE Rabbit Meat. https://www.tridge.com/intelligences/rabbit-meat.

[3] TRIDGE Rabbit Meat—Global Exports and Top Exporters. https://www.tridge.com/intelligences/rabbit-meat/export.

[4] Vega M.D., Peña A.I., Gullón J., Prieto C., Barrio M., Becerra J.J., Herradón P.G., Quintela L.A. (2008). Sex Ratio in Rabbits Following Modified Artificial Insemination. Anim. Reprod. Sci..

[5] Siddiqui S.A., Gerini F., Ikram A., Saeed F., Feng X., Chen Y. (2023). Rabbit Meat—Production, Consumption and Consumers’ Attitudes and Behavior. Sustainability.

[6] Van Ba H., Hwang I., Jeong D., Touseef A. (2012). Principle of Meat Aroma Flavors and Future Prospect. Latest Res. Qual. Control.

[7] Shi Y., Wang X., Borhan M.S., Young J., Newman D., Berg E., Sun X. (2021). A Review on Meat Quality Evaluation Methods Based on Non-Destructive Computer Vision and Artificial Intelligence Technologies. Food Sci. Anim. Resour..

[8] Daszkiewicz T., Gugołek A. (2020). A Comparison of the Quality of Meat from Female and Male Californian and Flemish Giant Gray Rabbits. Animals.

[9] Xie Y.J., He Z.F., Zhang E., Li H.J. (2016). Characterization of Key Volatile Odorants in Rabbit Meat Using Gas Chromatography Mass Spectrometry with Simultaneous Distillation Extraction. World Rabbit. Sci..

[10] Federation of Veterinarians of Europe (2017). FVE Position on Killing Unwanted Offspring in Farm Animal Production.

[11] Garnsworthy P.C. (2018). Reducing the Environmental Impact of Animal Production. Lat. Am. Arch. Anim. Prod..

[12] Balzani A., Aparacida Vaz do Amaral C., Hanlon A. (2021). A Perspective on the Use of Sexed Semen to Reduce the Number of Surplus Male Dairy Calves in Ireland: A Pilot Study. Front. Vet. Sci..

[13] Álvarez-Gallardo H., Kjelland M.E., Pérez-Martínez M., Villaseñor-González F., Romo-García S. (2022). Evaluation of Novel SexedULTRA-4M Technology for in Vitro Bovine Embryo Production. Anim. Reprod..

[14] Umehara T., Tsujita N., Shimada M. (2019). Activation of Toll-like Receptor 7/8 Encoded by the X Chromosome Alters Sperm Motility and Provides a Novel Simple Technology for Sexing Sperm. PLoS Biol..

[15] EMLAB Genetics EMLAB Genetics. Product Gallery. https://www.emlabgenetics.com/shop.

[16] ABS Global Sexcel® Sexed Genetics. https://www.absglobal.com/au/dairy/sexcel-sexed-genetics/.

[17] Bradley M.P. (1989). Immunological Sexing of Mammalian Semen: Current Status and Future Options. J. Dairy Sci..

[18] Yadav S.K., Gangwar D.K., Singh J., Tikadar C.K., Khanna V.V., Saini S., Dholpuria S., Palta P., Manik R.S., Singh M.K. (2017). An Immunological Approach of Sperm Sexing and Different Methods for Identification of X- and Y-Chromosome Bearing Sperm. Vet. World.

[19] Xie Y., Xu Z., Wu Z., Hong L. (2020). Sex Manipulation Technologies Progress in Livestock: A Review. Front. Vet. Sci..

[20] Johnson L.A. (1995). Sex Preselection by Flow Cytometric Separation of X and Y Chromosome-Bearing Sperm Based on DNA Difference: A Review. Reprod. Fertil. Dev..

[21] Lush J.L. (1925). The Possibility of Sex Control by Artificial Insemination with Centrifuged Spermatozoa. J. Agric. Res..

[22] Bhattacharya B.C. (1962). Different Sedimentation Rates of X- and Y- Sperm and the Question of Arbitrary Sex Determination. Z. Wissenschartliche Zool..

[23] Bedford J.M., Bibeau A.M. (1967). Failure of Sperm Sedimentation to Influence the Sex Ratio of Rabbits. J. Reprod. Fertil..

[24] Beatty R.A. (1969). Genetic Content and Buoyant Density of Rabbit Spermatozoa. J. Reprod. Fertil..

[25] Stambaugh R., Buckley J. (1971). Association of the lactic dehydrogenase X4 isozyme with male-producing rabbit spermatozoa. Reproduction.

[26] Zavos P.M. (1985). Sperm Separation Attempts via the Use of Albumin Gradients in Rabbits. Theriogenology.

[27] Copello M., Perez A., Marquez S., Sansinena M., Copello M., Perez A., Marquez S., Sansinena M. (2011). 202 Sex Pre-Selection in Rabbits: An Attempt to Skew Offspring Sex through Percoll and Swim-Up Sperm Preparation Techniques. Reprod. Fertil. Dev..

[28] Hussein A.M.A. (2013). Effect of sperm selection by percoll and swim up techniques on the sex ratio of rabbit offspring. J. Anim. Poult. Prod..

[29] Koltzoff N.K., Schröder V.N. (1933). Artificial Control of Sex in the Progeny of Mammals. Nature.

[30] Bangham A.D. (1961). Electrophoretic Characteristics of Ram and Rabbit Spermatozoa. Proc. R. Soc. Lond. B Biol. Sci..

[31] Nevo A.C., Michaeli I., Schindler H. (1961). Electrophoretic Properties of Bull and of Rabbit Spermatozoa. Exp. Cell Res..

[32] Gordon M.J. (1957). Control of sex ratio in rabbits by electrophoresis of spermatozoa. Proc. Natl. Acad. Sci. USA.

[33] Sevinç A. (1968). Experiments on Sex Control by Electrophoretic Separation of Spermatozoa in the Rabbit. J. Reprod. Fertil..

[34] Unterberger F. (1932). Geschlechtsbestimmung und Wasserstoffionenkonzentration. DMW-Dtsch. Med. Wochenschr..

[35] Wakim P.E. (1972). Determining the Sex of Baby Rabbits by Ascertaining the PH of the Vagina of the Mother before Mating. J. Am. Osteopath. Assoc..

[36] Muehleis P.M., Long S.Y. (1976). The Effects of Altering the PH of Seminal Fluid on the Sex Ratio of Rabbit Offspring. Fertil. Steril..

[37] Burkov I.A. (1968). The Effect of Immunisation of Rabbits with Cock Semen on the Sex of the Progen. The Effect of Immunisation of Rabbits with Cock Semen on the Sex of the Progeny.

[38] Hancock R.J.T. (1978). Comparison of Effects of Normal Rabbit Sera and Anti-Cock Sperm Sera on Rabbit Sperm, Including Comparison of Effects on the Sex Ratio. Biol. Reprod..

[39] Zavos P.M. (1983). Preconception Sex Determination via Intra-Vaginal Administration of H-Y Antisera in Rabbits. Theriogenology.

[40] Hays F.A. (1918). The Influence of Excessive Sexual Activity of Male Rabbits. II. On the Nature of Their Offspring. J. Exp. Zool..

[41] D’Amato C., Hagen D., Dziuk P.J. (1979). The Lack of Effect of Ejaculate Sequence on Sex Ratio in Rabbits. Reproduction.

[42] Johnson L.A., Flook J.P., Hawk H.W. (1989). Sex Preselection in Rabbits: Live Births from X and Y Sperm Separated by DNA and Cell Sorting. Biol. Reprod..

[43] Garner D., Gledhill B., Pinkel D., Lake S., Stephenson D., Van Dilla M., Johnson L. (1983). Quantification of the X-and Y-Chromosome-Bearing Spermatozoa of Domestic Animals by Flow Cytometry. Biol. Reprod..

[44] Johnson L.A. (1997). Advances in Gender Preselection in Swine. J. Reprod. Fertil. Suppl..

[45] Benedict R.C., Schumaker V.N., Davies R.E. (1967). The Buoyant Density of Bovine and Rabbit Spermatozoa. J. Reprod. Fertil..

[46] Kaneko S., Oshio S., Kobayashi T., Iizuka R., Mohri H. (1984). Human X- and Y-Bearing Sperm Differ in Cell Surface Sialic Acid Content. Biochem. Biophys. Res. Commun..

[47] Bhattacharya B.C. (1958). Sex Control in Mammals. Z. Tierzuecht. Zuechtungsbiol..

[48] Schilling E. (1971). Sedimentation as an Approach to the Problem of Separating X- and Y-Chromosome-Bearing Spermatozoa. J. Anim. Sci..

[49] Lindahl P.E. (1971). Centrifugation as a means of separating X- and Y-chromosome-bearing spermatozoa. J. Anim. Sci..

[50] Quinlivan W.L., Preciado K., Long T.L., Sullivan H. (1982). Separation of Human X and Y Spermatozoa by Albumin Gradients and Sephadex Chromatography. Fertil. Steril..

[51] Kaneko S., Yamaguchi J., Kobayashi T., Iizuka R. (1983). Separation of Human X- and Y-Bearing Sperm Using Percoll Density Gradient Centrifugation. Fertil. Steril..

[52] Hedge U.C., Shastry P.R., Rao S.S. (1977). A Simple and Reproducible Method for Separating Y-Bearing Spermatozoa from Human Semen. Indian J. Med. Res..

[53] Branham J.M. (1970). Separation of rabbit semen into two populations of spermatozoa by centrifugation. Reproduction.

[54] Cui Z., Sharma R., Agarwal A. (2016). Proteomic Analysis of Mature and Immature Ejaculated Spermatozoa from Fertile Men. Asian J. Androl..

[55] Brahem S., Mehdi M., Elghezal H., Saad A. (2011). Semen Processing by Density Gradient Centrifugation Is Useful in Selecting Sperm with Higher Double-Strand DNA Integrity. Andrologia.

[56] Hoffmann D.S., Killian G.J. (1981). Isolation of Epithelial Cells from the Corpus Epididymidis and Analysis for Glycery Lphosphorylchol Ine, Sialic Acid, and Protein. J. Exp. Zool..

[57] Ishijima S.A., Okuno M., Mohri H. (1991). Zeta Potential of Human X- and Y-bearing Sperm. Int. J. Androl..

[58] Ainsworth C.J., Nixon B., Aitken R.J. (2011). The Electrophoretic Separation of Spermatozoa: An Analysis of Genotype, Surface Carbohydrate Composition and Potential for Capacitation. Int. J. Androl..

[59] Shettles L.B. (1970). Factors Influencing Sex Ratios. Int. J. Gynecol. Obstet..

[60] Park Y.J., Shin D.H., Pang W.K., Ryu D.Y., Rahman M.S., Adegoke E.O., Pang M.G. (2021). Short-Term Storage of Semen Samples in Acidic Extender Increases the Proportion of Females in Pigs. BMC Vet. Res..

[61] You Y.A., Kwon W.S., Rahman M.S., Park Y.J., Kim Y.J., Pang M.G. (2017). Sex Chromosome-Dependent Differential Viability of Human Spermatozoa during Prolonged Incubation. Hum. Reprod..

[62] Bennett D., Boyse E.A. (1973). Sex Ratio in Progeny of Mice Inseminated with Sperm Treated with H-Y Antiserum. Nature.

[63] Prasad S., Rangasamy S., Satheshkumar S. (2010). Sex Preselection in Domestic Animals-Current Status and Future Prospects. Vet. World.

[64] Sills E.S., Kirman I., Colombero L.T., Hariprashad J., Rosenwaks Z., Palermo G.D. (1998). H-Y Antigen Expression Patterns in Human X- and Y-Chromosome-Bearing Spermatozoa. Am. J. Reprod. Immunol..

[65] Sumner A.T., Robinson J.A., Evans H.J. (1971). Distinguishing between X, Y and YY-Bearing Human Spermatozoa by Fluorescence and DNA Content. Nat. New Biol..

[66] Moruzzi J.F. (1979). Selecting a Mammalian Species for the Separation of X- and Y-Chromosome-Bearing Spermatozoa. Reproduction.

[67] Gledhill B.L., Pinkel D., Garner D.L. (1982). Identifying X- and Y-Chromosome-Bearing Sperm by DNA Content: Retrospective Perspectives and Prospective Opinions. https://www.osti.gov/biblio/5245674.

[68] Pinkel D., Lake S., Gledhill B.L., Van Dilla M.A., Stephenson D., Watchmaker G. (1982). High Resolution DNA Content Measurements of Mammalian Sperm. Cytom. J. Int. Soc. Anal. Cytol..

[69] Keeler K.D., Mackenzie N.M., Dresser D.W. (1983). Flow Microfluorometric Analysis of Living Spermatozoa Stained with Hoechst 33342. Reproduction.

[70] Johnson L.A. (1992). Gender Preselection in Domestic Animals Using Flow Cytometrically Sorted Sperm. J. Anim. Sci..

[71] Morrell J.M., Keeler K.D., Noakes D.E., Mackenzie N.M., Dresser D.W. (1988). Sexing of Sperm by Flow Cytometry. Vet. Rec..

[72] Johnson L.A., Welch G.R., Rens W. (1999). The Beltsville Sperm Sexing Technology: High-Speed Sperm Sorting Gives Improved Sperm Output for in Vitro Fertilization and AI. J. Anim. Sci..

[73] Rens W., Welch G.R., Johnson L.A. (1999). Improved Flow Cytometric Sorting of X- and Y-chromosome Bearing Sperm: Substantial Increase in Yield of Sexed Semen. Mol. Reprod. Dev..

[74] Garner D.L., Evans K.M., Seidel G.E. (2013). Sex-Sorting Sperm Using Flow Cytometry/Cell Sorting. Spermatogenes. Methods Protoc..

[75] Rens W., Welch G.R., Johnson L.A. (1998). A Novel Nozzle for More Efficient Sperm Orientation to Improve Sorting Efficiency of X and Y Chromosome-Bearing Sperm. Cytometry.

[76] Garner D.L., Seidel G.E. (2008). History of Commercializing Sexed Semen for Cattle. Theriogenology.

[77] Johnson L. (1992). Method to Preselect the Sex of Offspring. U.S. Patent.

[78] Buchanan K., Sharpe J., Michael K. (2018). Nozzle Assembly for a Flow Cytometer. U.S. Patent.

[79] Evans K. (2010). High Resolution Flow Cytometer. U.S. Patent.

[80] Cogent UK Ultraplus. https://www.cogentuk.com/sexed-ultra-1.

[81] Brito L., Vishwanath R., Heuer C., Evans K. (2019). Bovine Sexed Semen Production and Utilization. Clin. Theriogenol..

[82] Mastergen Ltd SexedUltraTM Semen. https://mastergen.com/factsheets/sexedultra-semen/.

[83] González-Marín C., Góngora C.E., Moreno J.F., Vishwanath R. (2021). Small Ruminant SexedULTRA^TM^ Sperm Sex-Sorting: Status Report and Recent Developments. Theriogenology.

[84] Fenner G.P., Johnson L.A., Hruschka W.R., Bolt D.J. (1992). Two-Dimensional Electrophoresis and Densitometry Analysis of Solubilized Bovine Sperm Plasma Membrane Proteins Detected by Silver Staining and Radioiodination. Arch. Androl..

[85] Rahman M.S., Pang M.G. (2020). New Biological Insights on X and Y Chromosome-Bearing Spermatozoa. Front. Cell Dev. Biol..

[86] Yeh Y.-C., Yang V.-C., Huang S.-C., Lo N.-W. (2005). Stage-Dependent Expression of Extra-Embryonic Tissue-Spermatogenesis-Homeobox Gene 1 (ESX1) Protein, a Candidate Marker for X Chromosome-Bearing Sperm. Reprod. Fertil. Dev..

[87] Quelhas J., Santiago J., Matos B., Rocha A., Lopes G., Fardilha M. (2021). Bovine Semen Sexing: Sperm Membrane Proteomics as Candidates for Immunological Selection of X- and Y-Chromosome-Bearing Sperm. Vet. Med. Sci..

[88] Umehara T., Tsujita N., Zhu Z., Ikedo M., Shimada M. (2020). A Simple Sperm-Sexing Method That Activates TLR7/8 on X Sperm for the Efficient Production of Sexed Mouse or Cattle Embryos. Nat. Protoc..

[89] Neves F., Marques J.P., Areal H., Pinto-Pinho P., Colaço B., Melo-Ferreira J., Fardilha M., Abrantes J., Esteves P.J. (2022). TLR7 and TLR8 Evolution in Lagomorphs: Different Patterns in the Different Lineages. Immunogenetics.

[90] Nuri Science Inc Animal Sperm Sexing. http://www.nurisci.com/eng/.

[91] Heo Y.-T., Kim D.-G., Uhm S. (2018). Analysis of Sex Ratio on Bovine in vitro Fertilized Embryos Using Sex Determination Kit Treated Sperm. J. Embryo Transf..

[92] Agarwal A., Panner Selvam M.K., Baskaran S. (2020). Proteomic Analyses of Human Sperm Cells: Understanding the Role of Proteins and Molecular Pathways Affecting Male Reproductive Health. Int. J. Mol. Sci..

[93] Casares-Crespo L., Fernández-Serrano P., Viudes-de-Castro M.P. (2019). Proteomic Characterization of Rabbit (*Oryctolagus cuniculus*) Sperm from Two Different Genotypes. Theriogenology.

[94] Douglas C., Maciulyte V., Zohren J., Snell D.M., Ojarikre O.A., Ellis P.J.I., Turner J.M.A. (2020). Generating Single-Sex Litters: Development of CRISPR-Cas9 Genetic Tools to Produce All-Male Offspring. bioRxiv.

[95] Douglas C., Maciulyte V., Zohren J., Snell D.M., Mahadevaiah S.K., Ojarikre O.A., Ellis P.J.I., Turner J.M.A. (2021). CRISPR-Cas9 Effectors Facilitate Generation of Single-Sex Litters and Sex-Specific Phenotypes. Nat. Commun..

[96] Gamez S., Chaverra-Rodriguez D., Buchman A., Kandul N.P., Mendez-Sanchez S.C., Bennett J.B., Sánchez C.H.M., Yang T., Antoshechkin I., Duque J.E. (2021). Exploiting a Y Chromosome-Linked Cas9 for Sex Selection and Gene Drive. Nat. Commun..

[97] Zhang Z., Niu B., Ji D., Li M., Li K., James A.A., Tan A., Huang Y. (2018). Silkworm Genetic Sexing through w Chromosome-Linked, Targeted Gene Integration. Proc. Natl. Acad. Sci. USA.

[98] Galizi R., Hammond A., Kyrou K., Taxiarchi C., Bernardini F., O’Loughlin S.M., Papathanos P.A., Nolan T., Windbichler N., Crisanti A. (2016). A CRISPR-Cas9 Sex-Ratio Distortion System for Genetic Control. Sci. Rep..

[99] Yin L., Maddison L.A., Li M., Kara N., Lafave M.C., Varshney G.K., Burgess S.M., Patton J.G., Chen W. (2015). Multiplex Conditional Mutagenesis Using Transgenic Expression of Cas9 and SgRNAs. Genetics.

[100] Domínguez E., Moreno-Irusta A., Castex H.R., Bragulat A.F., Ugaz C., Clemente H., Giojalas L., Losinno L. (2018). Sperm Sexing Mediated by Magnetic Nanoparticles in Donkeys, a Preliminary In Vitro Study. J. Equine Vet. Sci..

[101] Zuidema D., Kerns K., Sutovsky P. (2021). An Exploration of Current and Perspective Semen Analysis and Sperm Selection for Livestock Artificial Insemination. Animals.

[102] Perry G.A., Walker J.A., Rich J.J.J., Northrop E.J., Perkins S.D., Beck E.E., Sandbulte M.D., Mokry F.B. (2020). Influence of Sexcel^TM^ (Gender Ablation Technology) Gender-Ablated Semen in Fixed-Time Artificial Insemination of Beef Cows and Heifers. Theriogenology.

[103] Faust M.A., Betthauser J., Storch A., Crego S. (2016). Effects for Fertility of Processing Steps of a New Technology Platform for Producing Sexed Sperm. J. Anim. Sci..

[104] De Luca A.C., Managó S., Ferrara M.A., Rendina I., Sirleto L., Puglisi R., Balduzzi D., Galli A., Ferraro P., Coppola G. (2014). Non-Invasive Sex Assessment in Bovine Semen by Raman Spectroscopy. Laser Phys. Lett..

[105] Ferrara M.A., Di Caprio G., Managò S., De Angelis A., Sirleto L., Coppola G., De Luca A.C. (2015). Label-Free Imaging and Biochemical Characterization of Bovine Sperm Cells. Biosensors.

[106] Rosenfeld C.S., Roberts R.M. (2004). Maternal Diet and Other Factors Affecting Offspring Sex Ratio: A Review. Biol. Reprod..

[107] Green M.P., Spate L.D., Parks T.E., Kimura K., Murphy C.N., Williams J.E., Kerley M.S., Green J.A., Keisler D.H., Roberts R.M. (2008). Nutritional Skewing of Conceptus Sex in Sheep: Effects of a Maternal Diet Enriched in Rumen-Protected Polyunsaturated Fatty Acids (PUFA). Reprod. Biol. Endocrinol..

[108] Alhimaidi A.R., Ammari A.A., Alghadi M.Q., Al Saiady M.Y., Amran R.A., Swelum A.A. (2021). Sex Preselection of Sٍheep Embryo by Altering the Minerals of Maternal Nutrition. Saudi J. Biol. Sci..

[109] Ainsworth C., Nixon B., Aitken R.J. (2005). Development of a Novel Electrophoretic System for the Isolation of Human Spermatozoa. Hum. Reprod..

[110] Fleming S.D., Ilad R.S., Griffin A.M.G., Wu Y., Ong K.J., Smith H.C., Aitken R.J. (2008). Prospective Controlled Trial of an Electrophoretic Method of Sperm Preparation for Assisted Reproduction: Comparison with Density Gradient Centrifugation. Hum. Reprod..

[111] Diasio R.B., Glass R.H. (1971). Effects of PH on the Migration of X and Y Sperm. Fertil. Steril..

[112] Johnson L.A., Welch G.R. (1999). Sex Preselection: High-Speed Flow Cytometric Sorting of X and Y Sperm for Maximum Efficiency. Theriogenology.

[113] Feugang J.M., Rhoads C.E., Mustapha P.A., Tardif S., Parrish J.J., Willard S.T., Ryan P.L. (2019). Treatment of Boar Sperm with Nanoparticles for Improved Fertility. Theriogenology.

[114] Bisla A., Honparkhe M., Srivastava N. (2022). A Review on Applications and Toxicities of Metallic Nanoparticles in Mammalian Semen Biology. Andrologia.

[115] Jahmani M.Y., Hammadeh M.E., Al Smadi M.A., Baller M.K. (2021). Label-Free Evaluation of Chromatin Condensation in Human Normal Morphology Sperm Using Raman Spectroscopy. Reprod. Sci..

[116] UniProt Oryctolagus Cuniculus (Rabbit)|Proteomes. https://www.uniprot.org/proteomes/UP000001811.

[117] Weber J., Peng H., Rader C. (2017). From Rabbit Antibody Repertoires to Rabbit Monoclonal Antibodies. Exp. Mol. Med..

[118] Sela-Culang I., Kunik V., Ofran Y. (2013). The Structural Basis of Antibody-Antigen Recognition. Front. Immunol..

[119] Dodd R.B., Wilkinson T., Schofield D.J. (2018). Therapeutic Monoclonal Antibodies to Complex Membrane Protein Targets: Antigen Generation and Antibody Discovery Strategies. BioDrugs.

[120] El-Attrouny M.M., Habashy W.S. (2020). Correlated Response on Litter Traits and Milk Yield in New Zeland White Rabbits Selected for Litter Size at Birth. Egypt. Poult. Sci. J..

[121] Lebas F., Coudert P., de Rochambeau H., Thébault R.G. (1997). The Rabbit: Husbandry, Health and Production, Volume 21.

[122] Associação Portuguesa de Cunicultura (ASPOC). http://aspoc.pt/wp/wp-content/uploads/2023/07/Loncun-302023.pdf.

[123] Pollesel M., Tassinari M., Frabetti A., Fornasini D., Cavallini D. (2020). Effect of Does Parity Order on Litter Homogeneity Parameters. Ital. J. Anim. Sci..

